# Optometrische Schulreihenuntersuchungen: Erste Ergebnisse eines Pilotprojekts zur logistischen Machbarkeit

**DOI:** 10.1007/s00347-021-01394-5

**Published:** 2021-05-03

**Authors:** Hakan Kaymak, Kai Neller, Saskia Funk, Achim Langenbucher, Berthold Seitz, Hartmut Schwahn

**Affiliations:** 1 Institut für Experimentelle Ophthalmologie, Universitätsklinikum des Saarlandes UKS, Homburg/Saar, Deutschland; 2 Internationale Innovative Ophthalmochirurgie, Breyer Kaymak Klabe Augenchirurgie, Düsseldorf, Deutschland; 3 Klinik für Augenheilkunde, Universitätsklinikum des Saarlandes UKS, Homburg/Saar, Deutschland

**Keywords:** Myopie, Schulkinder, Achslänge, Prävention, Augengesundheit, Myopia, Schoolchildren, Axial length, Prevention, Eye health

## Abstract

**Hintergrund:**

Wir stellen ein Projekt zur Verbesserung der Augengesundheit bei Schulkindern vor: Jährliche optometrische Reihenuntersuchungen mit Fokus auf die Früherkennung der Schulmyopie. Die logistische Machbarkeit wird am Beispiel eines Pilotprojektes an einem staatlichen Gymnasium in Nordrhein-Westfalen beleuchtet. Die erhobenen biometrischen Parameter tragen außerdem zur Erhebung epidemiologischer Daten bei.

**Material und Methoden:**

An organisierten Untersuchungstagen wurde bei den Schulkindern der 5. bis 7. Klassen (Alter 9 bis 16 Jahre) die objektive und subjektive Refraktion ermittelt, auf Auffälligkeiten im Binokularsehen getestet und der photopische und mesopische Pupillendurchmesser bestimmt. Mittels berührungsfreier Biometrie wurden die Hornhautradien, zentrale Hornhautdicke, Vorderkammertiefe, Linsendicke und die Achslänge der Augen gemessen. Mittels optischer Kohärenztomographie (OCT) wurde außerdem die zentrale Aderhautdicke der Augen bestimmt. Mit Fragebögen wurden die Sehgewohnheiten der Schulkinder erfragt.

**Ergebnisse:**

Im Herbst 2019 nahmen 274 Schulkinder (11,2 ± 1,2 Jahre) freiwillig an den Untersuchungen teil; 22 % (61) zeigten eine Myopie (sphärisches Äquivalent ≤ −0,50 dpt), hiervon waren 11 % (7) bisher unkorrigiert (unkorrigierter Fernvisus < 0,8); 8 % (5) der Schulkinder zeigten eine Zunahme der Myopie um mehr als −0,5 dpt verglichen mit ihrem aktuellen Brillenwert (Fernvisus mit Brillenwert < 0,8). Eine Schulklasse mit ca. 25 Kindern kann innerhalb von 2 Schulstunden optometrisch untersucht werden.

**Diskussion:**

Die Notwendigkeit der optometrischen Reihenuntersuchung ist objektiv gegeben, da insgesamt 4,4 % (12) myope Schulkinder identifiziert werden konnten, die aufgrund ihrer Brillenkorrektion einen Fernvisus von kleiner 0,8 aufwiesen. Durch die Ermittlung der Achslänge und der Einordnung dieses Wertes in Abhängigkeit des Alters in die Literatur kann das individuelle Myopierisiko abgeschätzt und Eltern und Kinder können sensibilisiert werden, um dem Missstand zu begegnen. Die geplanten Wiederholungsuntersuchungen werden genauere Aussagen zum Bulbuswachstum bei Schulkindern liefern.

## Abstract (en)

**Background:**

We present a pilot project to improve eye health in schoolchildren: annual optometric screening with a focus on early detection of school myopia. The logistical proof-of-concept is illustrated by way of a pilot project at a state high school in North Rhine-Westphalia. The collected biometric parameters also contribute to the collection of epidemiological data.

**Material and methods:**

On organized examination days objective and subjective refractions of schoolchildren in grades 5–7 (ages 9–16 years) were determined, children were tested for abnormalities in binocular vision and the photopic and mesopic pupil diameters were determined. Noncontact biometry was used to measure the corneal radius, central corneal thickness, anterior chamber depth, lens thickness and axial length of the eyes. Optical coherence tomography (OCT) was used to also determine the central choroidal thickness of the eyes. Questionnaires were employed to inquire about the visual habits of the schoolchildren.

**Results:**

In the fall of 2019 a total of 274 schoolchildren (11.2 ± 1.2 years) voluntarily participated in the examinations: 22% (61) showed myopia (spherical equivalent ≤ −0.50 D), of which 11% (7) were previously uncorrected (uncorrected distance visual acuity < 0.8). Of the schoolchildren 8% (5) showed an increase in myopia of more than −0.5 D compared to their actual spectacle values (distance visual acuity with ophthalmic lens < 0.8). A class of about 25 pupils can be examined within 2 regular periods.

**Conclusion:**

There is an actual need for optometric screening because a total of 4.4% (12) myopic schoolchildren could be identified who had a distance visual acuity of less than 0.8 even with the correction. By determining the axial length and classifying this value depending on age in the literature can help to predict the individual risk of myopia and to raise awareness among parents and children to address this problem. The planned repetition of these examinations enables a better understanding of eye growth in schoolchildren.

Gutes Sehen spielt eine große Rolle für die physische und intellektuelle Entwicklung eines Kindes [5]. Im Schulalltag ist ein guter Fernvisus die Voraussetzung zum Lesen von der Tafel, ein problemloser Blickwechsel zwischen Ferne und Nähe ist wichtig, um dem Unterricht gut zu folgen, und ein guter Visus in der Nähe ist notwendig für problemloses Lesen und Schreiben. Auffälligkeiten im Binokularsehen und refraktive Fehler können dazu führen, dass die geforderten Aufgaben nur mangelhalft bearbeitet werden können, was einen erheblichen Einfluss auf die Schulentwicklung eines Kindes nehmen kann. Problematisch dabei ist, dass viele Kinder diesen Sehfehler von selbst nicht erkennen oder stark unterschätzen und dies von Eltern und Lehrkräften daher oft unbemerkt bleibt und folglich nicht behandelt wird.

Der Zusammenhang zwischen Prävalenz der Myopie bei Schulkindern und Anzahl der absolvierten Schuljahre ist bekannt [2, 9] – je höher der angestrebte Bildungsabschluss, desto höher die Anzahl myoper Schüler und Studenten [8]. Die in der Schulzeit erworbene Kurzsichtigkeit wird als Schulmyopie bezeichnet. Diese kritische Entwicklung beginnt zwischen dem 5. und 10. Lebensjahr [17]. Es gibt deutliche Hinweise, dass die aktuelle Seherfahrung (Lesen, Naharbeit und wenig Zeit im Freien) die Entstehung und Progression der Myopie beeinflusst [9]. Eine Schulmyopie ist primär durch solche Umweltfaktoren bedingt. Unterstützt wird diese These durch die Ergebnisse einer Metaanalyse, wonach das Bulbuswachstum durch eine lichtabhängige Signalkaskade von der Netzhaut zur Sklera getriggert wird [13]. Auch der in Asien beobachtete übermäßige Anstieg der Zahl kurzsichtiger Kinder und Jugendlichen ist hierauf zurückzuführen, da sich viele asiatische Länder in den letzten Jahrzehnten in Industrieländer entwickelt haben mit entsprechender Entwicklung des Bildungssystems [3, 7].

Mit hohen Myopiegraden (< −6 dpt) sind im Erwachsenenalter erhöhte Risiken ernsthafter Augenerkrankungen wie Netzhautablösung, Glaukom, früher Katarakt, myope Makulopathie und myope choroidale Neovaskularisation (CNV) verbunden [14]. Um der Entwicklung einer hohen Myopie vorzubeugen, gibt es inzwischen spezielle Brillengläser [6, 11], multifokale Kontaktlinsen [16] sowie die pharmakologische Therapie mit Atropin [1]. Zudem kann der regelmäßige Aufenthalt im Freien bei Tageslicht die Entwicklung einer Myopie bei Kindern hemmen [10].

Solche Maßnahmen können allerdings nur greifen, wenn Kinder und Jugendliche mit dem Risiko einer starken Myopieprogression bereits frühzeitig identifiziert werden. Aus epidemiologischen Normdaten kann mit den aktuell erhobenen Biometriedaten, insbesondere der Bulbuslänge (Achslänge), und der Refraktion das individuelle Risiko einer leichten, mittelgradigen oder hohen Myopie abgeschätzt werden [15]. Diese Information könnte Kinder und deren Eltern frühzeitig befähigen und motivieren, myopiehemmende Maßnahmen zu ergreifen.

Wir schlagen die Installation einer entsprechenden Gruppenprophylaxe für Myopie entsprechend der an den Schulen und Kindergärten gut etablierten zahnmedizinischen Gruppenprophylaxe vor und berichten über unsere ersten Erfahrungen mit einem Pilotprojekt einer solchen Reihenuntersuchung. Schüler der 5. bis 7. Klasse eines Gymnasiums wurden optometrisch untersucht, und die Eltern wurden über den Refraktionsstatus, sonstige Auffälligkeiten und das individuelle Myopierisiko ihres Kindes informiert. In den geplanten jährlichen Folgemessungen werden die Entwicklung der Myopie, die Emmetropisierung sowie eine Entwicklung des individuellen Myopierisikos bei den Schulkindern zu beobachten sein. Die im Rahmen dieses Pilotprojektes zusätzlich erhobenen Werte sollen in Zukunft dazu beitragen, Normdaten für das Myopiemanagement für den europäischen Raum bereitzustellen.

## Material und Methoden

Alle Untersuchungen wurden mit Zustimmung der Ethik-Kommission des Universitätsklinikums Jena (Nr.: 2019/1520), im Einklang mit nationalem Recht sowie gemäß der Deklaration von Helsinki von 1975 durchgeführt. Von allen beteiligten Schulkindern und deren Eltern liegt eine Einverständniserklärung vor.

### Planung und Durchführung der ersten optometrischen Untersuchung

Von September bis November 2019 fanden die ersten Reihenuntersuchungen der 5. bis 7. Jahrgangsstufen an einem staatlichen Gymnasium im Raum Düsseldorf statt. Zu den optometrischen Messungen gaben die Eltern und die Schulkinder nach Aufklärung im Vorfeld ihr schriftliches Einverständnis. Den Eltern wurde ein Anamnesebogen ausgeteilt, welcher die Ethnie, das Geschlecht, das Alter, aber auch die Gewohnheiten der Kinder: Zeit im Freien, Zeit für Naharbeit, Zeit am Smartphone, abfragt. Die Messungen selbst fanden in der Aula der Schule an insgesamt 4 Tagen statt. Für jede Schulklasse (jeweils ca. 24 Kinder) wurde eine Doppelstunde (90 min) eingeplant. Der Aufwand für das Auf- und Abbauen der Instrumente wurde mit jeweils 1 h veranschlagt, sodass sich für das Messteam ein Arbeitspensum von 8 h/Tag ergab. Es waren 8 Mitarbeiter an jedem Messtag vor Ort.

**Figure 1:**
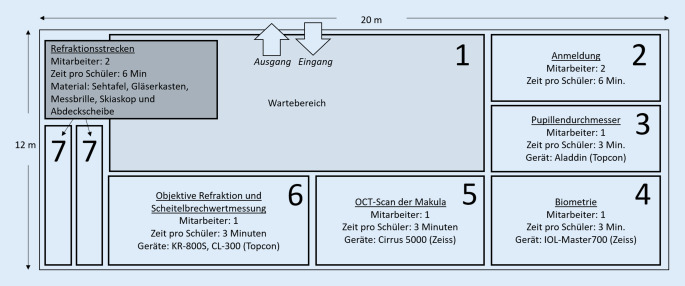

Die Abb. 1 zeigt schematisch den Aufbau. Zunächst nahmen alle Schüler im Wartebereich (1) Platz, wurden dann anonym mit einer zuvor zugeteilten Probandennummer aufgerufen und gingen mit der ebenfalls anonymisierten Einverständniserklärung ihrer Eltern zur Anmeldung (2). Hier mussten sie selbst nach einem Aufklärungsgespräch ihr Einverständnis geben. Die Schüler erhielten einen Laufzettel und wurden angewiesen, sich selbstorganisiert an den Stationen (3) bis (6) anzustellen; die Reihenfolge war frei wählbar. Die Station (6): objektive Refraktion, musste vor der Station (7): subjektive Refraktion, besucht werden.

Zur Bestimmung der Refraktion waren die Kinder im Vorfeld informiert, am Tag der Untersuchung keine Kontaktlinsen zu tragen. An Station (6) wurden bei jedem Kind eine objektive Refraktion (KR-800S, Topcon) (Topcon, Tokyo, Japan) und bei Brillenträgern zusätzlich mittels Scheitelbrechwert (CL-300, Topcon) (Topcon, Tokyo, Japan) die aktuellen Brillenwerte der getragenen Sehhilfe bestimmt (Station 6). Die subjektive Refraktion wurde dann zusätzlich bestimmt, wenn bei mindestens 1 Auge ein sphärischer Wert < 0 dpt oder > 0,5 dpt und/oder Astigmatismus ≥ 1 dpt gemessen wurde oder der erhobene Visus einen Unterschied zwischen den Augen von mehr als 2 Visusstufen aufwies oder der erhobene Visus auf mindestens 1 Auge unter 0,8 lag. Die subjektive Refraktion erfolgte durch erfahrene Optometristen an einer herkömmlichen, hintergrundbeleuchteten Sehzeichentafel (Fa. Good-Lite, USA) (Good-Lite, Elgin, IL, USA). Zunächst wurden die in der objektiven Refraktion ermittelten Werte in die Messbrille UB4 (Fa. Oculus Optikgeräte GmbH) (Oculus Optikgeräte GmbH, Wetzlar, Deutschland) eingesetzt. Um Zeit zu sparen, erfolgte zunächst ein grober Abgleich der Fernpunktrefraktion mittels Skiaskop (Beta 200, Fa. Heine) (Heine, Gilching, Deutschland) und Abgleichleiste. Die subjektive Refraktion galt als bestimmt, wenn ein Visus von ≥ 1,0 erzielt wurde oder durch das Vorhalten von sphärischen −0,25 dpt kein Visusanstieg zu verzeichnen war. Wenn kein Kriterium zur subjektiven Refraktion erfüllt war, wurde an Station (7) nur mittels Cover- und Uncover-Tests auf Auffälligkeiten im Binokularsehen geprüft.

An Station (4) wurde mittels Biometer (IOL Master 700, Fa. Zeiss) (Zeiss, Oberkochen, Deutschland) an beiden Augen die Achslänge bestimmt.

### Statistische Analyse

Die Boxplots, Tortenendiagramme und Bland-Altman-Diagramme wurden mit Matlab (Version 2020b, Fa. The MathWorks Inc) (MathWorks, Natick, MA, USA) erzeugt. Unter Verwendung der Statistiksoftware R (Version 3.6.0, The R Foundation) (R Foundation, Wien, Österreich) wurden mittels multivariabler logistischer Regression mögliche Risikofaktoren der Myopie unadjustiert und volladjustiert betrachtet.

## Ergebnisse

### Gefundene Prävalenz der Fehlsichtigkeit

**Figure 2:**
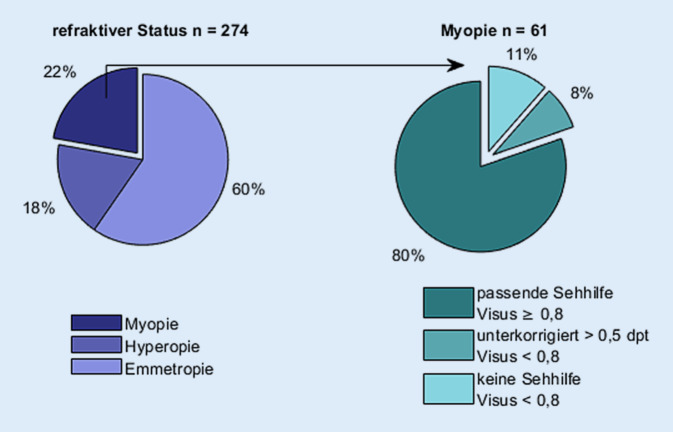

Die 5. bis 7. Jahrgangsstufen des Gymnasiums umfassten 13 Schulklassen mit insgesamt 304 Schulkinder, von denen 274 Schulkinder an den Reihenuntersuchungen teilnahmen (Teilnahmequote 90 %), wobei 13 Kinder aus einer Schulklasse nicht an den Untersuchungen teilnahmen, sodass sich für die anderen 12 Klassen eine Teilnahmequote von 94 % ergibt. Von den insgesamt 274 untersuchten Schulkindern (70 % männlich) der Altersgruppe 9 bis 16 Jahre (11,2 ± 1,1 Jahre) waren 22 % (61) der Schulkinder myop, 60 % (163) emmetrop und 18 % (50) hyperop (Abb. 2). Die Unterteilung in Hyperopie, Emmetropie und Myopie erfolgte nach der Berechnung des sphärischen Äquivalents (SÄ): Hyperopie: SÄ > +0,50 dpt, Emmetropie: −0,50 < SÄ ≤ +0,5 dpt, Myopie: SÄ ≤ −0,50 dpt. Von den aufgefundenen 61 myopen Schulkindern waren 7 Schüler bisher unkorrigiert (unkorrigierter Fernvisus < 0,8), und bei weiteren 5 Schülern zeigte sich in der Refraktion eine Zunahme des myopen Refraktionsfehlers um mehr als 0,5 dpt verglichen mit ihrem jeweiligen Brillenwert (Fernvisus mit Brillenwert < 0,8) (Abb. 2). Ein Kind zeigte einen Nystagmus, dieser war bereits ärztlich bekannt. Bei diesem Kind konnten aufgrund fehlender Fixation die Biometrie und der Pupillendurchmesser nicht ausgewertet werden. Mit Ausnahme dieses Kindes konnte bei keinem weiteren Kind eine Auffälligkeit im Binokularsehen festgestellt werden.

**Figure 3:**
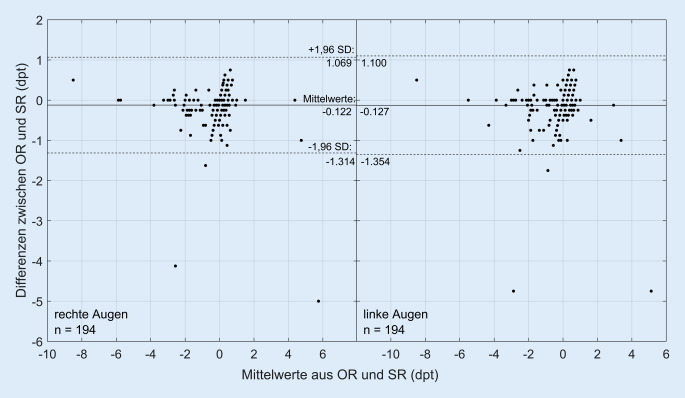

Bei insgesamt 194 der 274 untersuchten Kinder wurde eine subjektive Refraktion durchgeführt. In Abb. 3 sind in Form von Bland-Altman-Diagrammen (rechtes und linkes Auge) die Refraktionswerte der objektiven und subjektiven Refraktion dargestellt. Ein negativer Wert auf der y‑Achse gibt an, dass in der objektiven Refraktion ein mehr negativer Wert gemessen wurde als in der subjektiven Refraktion. Bei 2 Kindern (beide 11 Jahre alt) unterschieden sich die Werte zwischen objektiver und subjektiver Refraktionsbestimmung um mehr als 4 dpt. Beide Kinder hatten laut der objektiven Refraktion einen Fernvisus von 0,1, zeigten aber in der subjektiven Refraktion einen Fernvisus von 1,0. Die mittleren Refraktionswerte der untersuchten Kinder liegen bei −0,29 ± 1,38 dpt und −0,29 ± 1,32 dpt (objektive Refraktion, rechte und linke Augen) und bei −0,17 ± 1,44 dpt und −0,16 ± 1,34 dpt (subjektive Refraktion, rechte und linke Augen).

**Figure 4:**
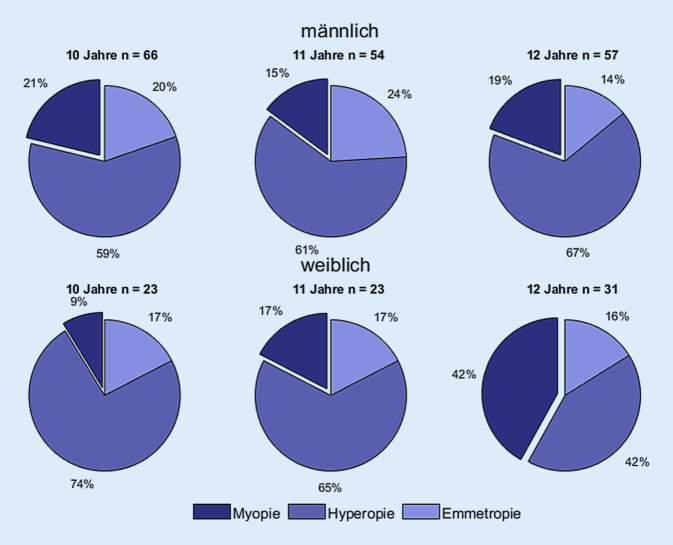

Die Abb. 4 zeigt die Unterteilung der Fehlsichtigkeit in Geschlecht und Altersgruppe (10 bis 12 Jahre). Für die jüngeren und älteren Altersgruppen sind zur Auswertung zu wenig Daten vorhanden.

### Auswertung des Anamnesebogens

Insgesamt lagen von 266 der 274 untersuchten Kinder die Anamnesebögen zur Auswertung vor. Bei 8 Schulkindern wurde kein ausgefüllter Fragebogen zurückerhalten.

Die Analyse der Daten zeigt, dass ein höheres Alter mit einer höheren Prävalenz der Myopie assoziiert ist, wohingegen das Geschlecht nicht mit einer höheren Myopieprävalenz assoziiert ist. Es zeigt sich ein inverser Zusammenhang zwischen dem Kriterium „tägliche Zeit draußen“ und der Prävalenz der Myopie, welcher auch nach Adjustierung der Daten für Alter und Geschlecht bestehen bleibt. Die weiteren Kriterien „tägliche Zeit am Smartphone“ und „tägliche Lesezeit“ zeigen im multivariablen Modell keinen Zusammenhang zur Myopie (Tab. 1).

**Table 1:** 

	Unadjustiert			Adjustiert		
	Odds Ratio	95 %-Konfidenzintervall für Odds Ratio	*p*-Wert	Odds Ratio	95%-Konfidenzintervall	*p*-Wert
Alter (Jahr)	1,58	(1,21; 2,09)	< 0,001	1,65	(1,24; 2,23)	< 0,001
Geschlecht (männlich)	0,71	(0,39; 1,31)	0,27	0,85	(0,45; 1,63)	0,62
**Mögliche Risikofaktoren**						
*Tägliche Lesezeit*						
< 1 h	Ref.	–	0,87	Ref.	–	0,79
1–2 h	1,18	(0,63; 2,21)		0,97	(0,48; 1,92)	
> 2 h	1,06	(0,42; 2,47)		1,36	(0,50; 3,43)	
*Tägliche Zeit draußen*						
< 1 h	3,10	(1,41; 6,85)	< 0,05	3,48	(1,49; 8,23)	< 0,05
1–2 h	1,22	(0,63; 2,39)		1,49	(0,50; 3,43)	
> 2 h	Ref.	–		Ref.	–	
*Tägliche Zeit am Smartphone*						
< 1 h	Ref.	–	0,55	Ref.	–	0,76
1–2 h	0,85	(0,44; 1,61)		0,78	(0,39; 1,53)	
> 2 h	1,33	(0,61; 2,82)		0,95	(0,40; 2,18)	

Volladjustierte Analysen sind adjustiert für Alter und Geschlecht und beinhalten alle Risikofaktoren (tägliche Lesezeit, tägliche Zeit draußen und tägliche Zeit am Smartphone)

**Figure 5:**
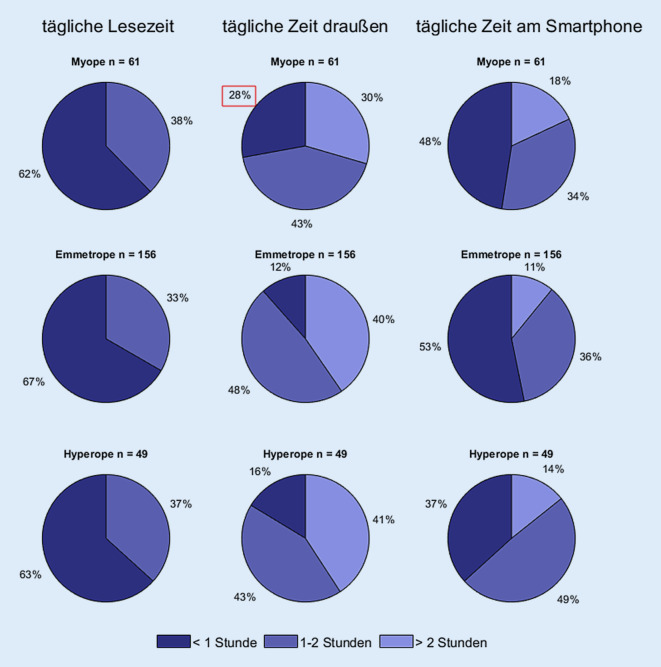

In Abb. 5 sind die Ergebnisse des Anamnesebogens zu den Risikofaktoren für eine Myopie dargestellt. Es zeigt sich, dass 28 % der befragten myopen Kinder bei dem Kriterium „tägliche Zeit draußen“ angaben, sich täglich weniger als 1 h im Freien aufzuhalten (rot gekennzeichnet).

## Diskussion

### Gesundheitlicher Mehrwert für die Schulkinder

Im Rahmen dieser ersten Messserie unserer Pilotstudie zu optometrischen Schulreihenuntersuchungen konnte die 5. bis 7. Jahrgangsstufe eines Gymnasiums erfolgreich vermessen werden. Der zeitliche Aufwand für die optometrischen Messungen betrug jeweils 2 Schulstunden (90 min). Der Zeitaufwand pro Kind liegt bei etwa 15 min. Der gesundheitliche Mehrwert ist hoch, da insgesamt 12 myope Schüler (4,5 % der untersuchten Gruppe) identifiziert werden konnten, die mit ihrer aktuellen Korrektion nur einen Fernvisus von kleiner 0,8 erreichten. Besondere Aufmerksamkeit in den Folgemessungen sollte hinsichtlich der Schulmyopie auf Kinder gelegt werden, welche aktuell noch als emmetrop eingestuft werden. Wie bei Zadnik et al. [19] beschrieben, werden Kinder, welche in der fünften Klasse bereits ein sphärisches Äquivalent von +0,25 dpt haben, in der sechsten Klasse bereits myop sein. Die regelmäßige Refraktionsmessung liefert somit wichtige Hinweise darauf, wann und ob ein Kind myop wird. Hinsichtlich der Prävalenz der Myopie bei den Jungen (16,7 %) und Mädchen (23,4 %) liegen die Werte über denen der KiGGS-Studie für die Altersgruppe 10 bis 13 Jahre (Jungen: 10–15 %, Mädchen: 15–20 %) [12].

Das Erkennen von Sehstörungen ist in den vom Gemeinsamen Bundesausschuss (GBA) festgelegten Richtlinien über die Früherkennung von Krankheiten bei Kindern (Kinder-Richtlinien) in den Untersuchungen U7 bis U9 festgelegt. Der vorgesehene Untersuchungszeitraum für die U9-Untersuchung endet mit dem 66. Lebensmonat (5,5 Jahre). In der Literatur ist beschrieben, dass sich eine Schulmyopie ab dem 6. Lebensjahr entwickeln kann [17]. Die auf die U‑Untersuchungen folgenden Jugendgesundheitsuntersuchungen (J1 und J2) verfolgen das Ziel der „Früherkennung von Erkrankungen, die die körperliche, geistige und soziale Entwicklung in nicht geringfügigem Maße gefährden. [4]“. Bei diesen Untersuchungen, welche zwischen dem 12. und 17. Lebensjahr stattfinden, kann das Erheben des Visus (wenn vorhanden mit Sehhilfe) helfen, eine unentdeckte Myopie bzw. die Progression der Myopie aufzudecken, welche dann genauer durch einen qualifizierten Augenarzt oder Optometristen untersucht wird. Hier sollte unter Vollkorrektion der Visus erhoben werden, da nur so über Jahre bei wiederkehrenden Messungen eine Myopieprogression dokumentiert werden kann. Dieser Weg wäre mit weniger Personalaufwand verbunden und womöglich politisch eher durchsetzbar. Allerdings war es das Ziel der im Rahmen dieser Pilotstudie durchgeführten Untersuchungen, einerseits die Grenzen des Machbaren auszutesten und zusätzlich auch im Hinblick auf die Generierung epidemiologischer Daten möglichst exakte und vollständige Messungen durchzuführen. Insbesondere das Messen der Achslänge zur Dokumentation der Myopieprogression und Abschätzung des individuellen Myopierisikos spielt eine zentrale Rolle.

### Refraktionsbestimmung in der Reihenuntersuchung

Die in Abb. 3 in Form eines Bland-Altman-Diagramms dargestellten Refraktionswerte zeigen die leichte Tendenz eines Autorefraktometers, die Refraktion mit einem höher negativen Wert im Vergleich zur subjektiven Refraktion anzugeben. Grund hierfür ist primär die Akkommodationsfähigkeit der Kinder. Insbesondere bei der Datenerhebung zur Schulmyopie, der genauen Bestimmung des Eintritts der Myopie und der Myopieprogression kann dies zu einer Fehlinterpretation der Daten führen. Die Akkommodation bei Kindern kann zwar mittels Zykloplegika ausgeschaltet werden, dies darf allerdings nur unter der Anwesenheit eines Augenarztes erfolgen und setzt das Einverständnis der Erziehungsberechtigten voraus. Die Wirkung der Zykloplegika ist verbunden mit einer Mydriasis, sodass die Kinder nach solchen Untersuchungen nicht mehr in der Lage wären, mit guten Sehleistungen am Schulunterricht teilzunehmen. Folglich kommt die Gabe von Zykloplegika für die Refraktionsbestimmung nicht infrage. Vielmehr sollte durch erfahrene Optometristen über sorgfältige Beobachtung und verhaltensleitende Maßnahmen die Tendenz zur Akkommodation bei der Messung unterdrückt werden.

### Individuelles Myopierisiko

In der Literatur ist ein eindeutiger Zusammenhang zwischen der täglichen Lesezeit, sowie der täglichen Zeit draußen und der Entwicklung einer Myopie bei Kindern beschrieben [2, 9, 10]. Bezogen auf das Kriterium „tägliche Zeit draußen“, konnte dieser Zusammenhang auch in unseren Untersuchungen belegt werden. In der Literatur ist eine Formel zum Abschätzen des Myopierisikos anhand der gemessene täglichen Naharbeit („dioptic hours“) gegeben [18]. Die tägliche Zeit im Freien ist ein aussagekräftiger Faktor für das individuelle Myopierisiko. Liegt diese unter 1 h, ist mit einer signifikant höheren Myopie zu rechnen. Wie auch von Schuster et al. [12] gezeigt wurde, besteht kein Zusammenhang zwischen der Nutzung eines Smartphones und der Entwicklung einer Myopie. Entgegen den Daten von Schuster et al. [12], zeigt sich kein Zusammenhang zwischen der Myopie und dem Geschlecht, was vermutlich mit der ungleichen Verteilung der Geschlechter in unserer Stichprobe zusammenhängt.

In dem Anamnesefragebogen wurden die Eltern über das Verhalten ihres Kindes befragt, wobei aber bei der Beantwortung auch die soziale Erwünschtheit einen Einfluss hat. Dieser Einfluss wurde vorliegend aber nicht weiter untersucht.

## Fazit für die Praxis

- Der Mehrwert für die Schulkinder und damit die Notwendigkeit von optometrischen Reihenuntersuchungen an Schulen ist schon bereits wegen der gefundenen Zahl von nicht und nur schlecht korrigierten Schulkindern gegeben.
- Durch die Reihenuntersuchung können nicht nur bereits myope Kinder identifiziert werden, sondern auch frühzeitig individuelle Myopierisiken wie hohes Achslängenwachstum und Auffälligkeiten im Verhalten bemerkt werden.
- Schulreihenuntersuchungen schaffen ein Bewusstsein bei Schülern, Eltern und Lehrern über die Gefahren einer Myopie und die Notwendigkeit einer frühen Intervention.
- Über die Messung der gesamten Biometrie der Augen, der Aderhautdicke und der Pupillengröße wird eine gesonderte Publikation erstellt.
